# Long- and Short-Run Asymmetric Effects of Meteorological Parameters on Hemorrhagic Fever with Renal Syndrome in Heilongjiang: A Population-Based Retrospective Study

**DOI:** 10.1155/2024/6080321

**Published:** 2024-07-30

**Authors:** Yongbin Wang, Bingjie Zhang, Chenlu Xue, Peiping Zhou, Xinwen Dong, Chunjie Xu

**Affiliations:** ^1^ Department of Epidemiology and Health Statistics School of Public Health The First Affiliated Hospital Xinxiang Medical University, No. 601 Jinsui Road, Hongqi District, Xinxiang 453003, Henan, China; ^2^ Beijing Key Laboratory of Antimicrobial Agents/Laboratory of Pharmacology Institute of Medicinal Biotechnology Chinese Academy of Medical Sciences and Peking Union Medical College, Beijing 100050, China

## Abstract

Examining both long-term and short-term effects can enhance the precision and reliability of time series analysis. This study aimed to delve into the asymmetric effects of weather conditions on hemorrhagic fever with renal syndrome (HFRS) in the long and short terms and build a forecasting system. Data comprising monthly HFRS incidents and weather factors in Heilongjiang from January 2004 to December 2019 were extracted. Subsequently, the long- and short-term asymmetric impacts were examined using the autoregressive distributed lag (ARDL) and nonlinear ARDL (NARDL) models. Next, the samples were partitioned into training and testing subsets to evaluate the predictive potential of both models. From 2004 to 2019, HFRS exhibited a declining trend (average annual percentage change = −6.744%, 95% CI: −13.52%–0.563%) and a dual seasonal pattern, with a prominent peak in June and a secondary one in October–December. This study identified long-term asymmetric effects of rainfall (Wald long-run asymmetry (WLR) = 3.292, *p*=0.001), wind velocity (WLR = −3.271, *p*=0.001), and air pressure (WLR = −6.453, *p* < 0.001) on HFRS. Additionally, this study observed short-term asymmetric impacts of relative humidity (Wald short-run symmetry (WSR) = −1.547, *p*=0.001), rainfall (WSR = −1.984, *p*=0.049), and air pressure (WSR = −2.33, *p*=0.021) on HFRS. A unit increase in relative humidity, sunshine hours, and air pressure resulted in about 10.9%, 1.9%, and 13.6% decreases in HFRS, respectively; a unit decrease in relative humidity, rainfall, and sunshine hours led to about 6.7%, 1.8%, and 2% decreases in HFRS, respectively. When temperature increased and decreased by one unit, the HFRS incidence increased by 11.6% and 22.5%, respectively. HFRS also varied significantly with the positive and negative changes in differenced (D) temperature, D (relative humidity), D (wind velocity), D (rainfall), D (air pressure), and D (sunshine hours) at 0−3-month delays over the short term. The NARDL model exhibited notably lower error rates in forecasting compared to the ARDL model. Meteorological parameters affect HFRS in both the long and short term, often showing asymmetric effects. The NARDL model, capable of incorporating various weather parameters, proves to be valuable in predicting HFRS epidemic and guiding strategies for prevention and control.

## 1. Introduction

Hemorrhagic fever with renal syndrome (HFRS) is a rodent-borne zoonosis caused by hantaviruses [1]. When individuals are infected with these viruses, they often experience symptoms such as fever, chills, nausea, blurred vision, and acute renal failure [1]. HFRS is primarily reported in eastern Asia, particularly in China, Russia, and Korea [2]. China has the highest number of cases, accounting for about 90% of the global burden [3, 4]. Although there has been a significant reduction in HFRS incidence since the 1990s due to effective measures [3], it remains highly endemic in 28 out of 31 provinces in China. Each year, there are over 5,000 case notifications, with a fatality rate of 3%–10% [3, 5]. Since 2009, there has been a resurgence in HFRS incidence, posing a threat to public health [6]. To effectively control the spread of HFRS, it is crucial to examine how the potential determinants play roles and construct an early warning system.

There has been growing interest in understanding the impact of climatic factors on the transmission of communicable diseases as these variables are capable of influencing the growth and development of pathogenic agents, host population dynamics, and human behaviors [7], making them potential early warning indicators of disease epidemic [8]. Earlier work has documented a correlation between meteorological factors and HFRS [7, 9, 10, 11, 12, 13]. For example, Xiang et al. [7] indicated that a 1°C increase in maximum temperature, a 1 mm increase in precipitation, and a 1% increase in average relative humidity were linked to 1.6%, 0.2%, and 0.9% increases in HFRS in 19 cities based on a generalized estimating equation model. Wei et al. [9] found that a 1°C increase in temperature and a 1 mm increase in aggregate rainfall were associated with 5.54% and 0.08% declines in HFRS in Guangzhou based on a negative binomial multivariable regression. Huang et al. [10] suggested that there was a significant association of relative humidity at a 1-month lag (*β* = −0.01) and a 3-month lag (*β* = 0.01), together with maximum temperature at a 2-month lag (*β* = 0.08) with HFRS transmission based on an autoregressive integrated moving average (ARIMA). Lv et al. [12] identified a significant negative effect of heavy rainfall on HFRS in the Hantaan virus-dominant endemic area and a parabolic or linear association of temperature and relative humidity with HFRS in China based on a generalized additive model (GAM) and distributed lag nonlinear model (DLNM). Wang et al. [14] indicated that extremely low and high temperatures contributed to the increases in HFRS cases in Shandong using a GAM model. But there are several gaps in the current study findings. First, inconsistencies arise due to the use of different statistical analysis methods and variations in climate across various regions [7, 9, 10, 12, 15, 16, 17, 18]. Second, previous studies have predominantly focused on temperature, air pressure, rainfall, and humidity, neglecting the potential impacts of sunshine and wind [12, 19]. However, these six weather variables coexist, and their combined exposure may lead to intricate interactions between positive and negative changes, influencing HFRS dynamics. Third, a lack of consideration for autocorrelations among dependent variables has resulted in overestimations [20]. Lastly, there is a significant gap in understanding whether an increase or decrease in climatic variables results in different impacts on HFRS (called “asymmetry,” see the details of this effect in Supplementary Materials) and how changes in climatic factors affect HFRS transmission over both short and long terms. The short-term effect represents the immediate and transient impacts that meteorological variables exert on HFRS transmission within a relatively short-time frame. Additionally, this effect reveals how swiftly the system adjusts toward its long-term equilibrium after experiencing a disturbance or shock. Understanding these transient impacts is crucial for policymakers and decision-makers, as it provides insights into the immediate consequences of climate change on HFRS transmission [21]. While the long-term effect also called “cointegration,” which describes a state in which variables tend to revert to a stable relationship over time. This phenomenon reveals underlying trends, patterns, and relationships that drive the behavior of HFRS epidemics in the long term. By discerning these persistent influences, one can better understand the broader implications and impacts [22]. Therefore, investigating both effects can improve the accuracy and reliability of time series analysis, crucial for informing the development of public health policies.

To deal with the gaps above, the current study introduced a nonlinear autoregressive distributed lag (NARDL) in analyzing the relationship between meteorological variables and HFRS. This choice was motivated by several advantages, including its ability to capture both long- and short-term asymmetries of meteorological factors on HFRS transmission, its flexibility in handling the cointegration of variables even with limited data, its effectiveness in addressing endogeneity among meteorological factors, and its capability to automatically identify autocorrelations. Recognizing that Heilongjiang faces the highest risk among all HFRS-endemic provinces in China [6], the objectives of this study are twofold: (1) to examine the long- and short-term asymmetric relationships of climatic variables and HFRS in Heilongjiang using the NARDL model, and (2) to assess whether the NARDL model provides a more accurate estimation of the HFRS epidemic compared to the autoregressive distributed lag (ARDL) model. These findings may provide valuable insights into the complex interactions between weather conditions and HFRS, which is pivotal for developing scenario plans aimed at controlling climate-driven transmission of HFRS and contributes to enhancing local healthcare services and informing policy actions in response to the risks posed by HFRS transmission.

## 2. Materials and Methods

### 2.1. Study Area

Heilongjiang Province, situated in the northeastern part of China, lies between east longitude 121°11′−135°05′ and north latitude 43°26′−53°33′. It shares borders with Russia to the north and east, the Inner Mongolia Autonomous Region to the west, and Jilin Province to the south (*Supplementary 1*). Heilongjiang covers a total area of 473,000 km^2^, ranking the sixth area in China. The province features diverse landscapes, including mountains, plateaus, plains, and water bodies. Its climate exhibits distinct seasonal patterns: cold and dry springs, hot and rainy summers, flood-prone autumns, prolonged winters, and a brief frost-free period. Additionally, significant regional climate variations exist. As of the end of 2023, the permanent resident population in Heilongjiang stood at approximately 30.62 million.

### 2.2. HFRS Data

The monthly HFRS notification cases in Heilongjiang between 2004 and December 2019 were retrieved from the data center of China Public Health Science (https://www.phsciencedata.cn/). The corresponding population data were sourced from the Heilongjiang Statistical Yearbook 2022. All HFRS notification cases were confirmed following the diagnostic criteria issued by the Chinese Ministry of Health (http://www.nhc.gov.cn/zwgkzt/s9491/200802/39043.shtml). These confirmed cases were subsequently reported through the Notifiable Infectious Disease Surveillance System within 24 hr by authorized institutions and professionals. This ensures accurate monitoring and timely reporting of HFRS cases, contributing to effective public health management.

### 2.3. Meteorological Data

Monthly climatic parameters such as average temperature, average wind velocity, aggregate rainfall, average air pressure, aggregate sunshine hours, and average relative humidity were obtained from the National Meteorological Science Data Center (http://data.cma.cn/). Some stations may have missing data due to observation reasons, and therefore the monthly weather variables from China Meteorological Yearbook (2022) were also extracted. These additional data will enhance the comprehensiveness of the meteorological variables.

### 2.4. Statistical Analysis

According to the normality test results using the Shapiro–Wilk method, the data were expressed as mean ± standard deviation ($\overset{―}{x}\pm s$) (normal distribution) or median (Q_25_, Q_75_) (skewed distribution). We utilized the average annual percentage change and season index (which indicates the degree to which the epidemic level of HFRS during a specific period deviates from the average level. A value = 1 indicates that the epidemic level of HFRS for that period matches the average. A value >1 suggests that the epidemic level of HFRS is above the average, while a value <1 indicates that the epidemic level of HFRS is below the average) to elucidate the changing trends and seasonal patterns in HFRS incidence, respectively [23]. Furthermore, we investigated the relationship between weather variables and HFRS using Spearman's rank correlation (*r*_*s*_). If *r*_*s*_ > 0.9 or variance inflation factor (VIF) >10, it indicated the presence of multicollinearity among factors [9, 24]. However, at this juncture, these factors cannot be included in the same models with other weather variables to explore the independent effect.

The ARDL model has been considered as a baseline model to examine the short- and long-run dynamic associations between regressors and dependent variable because it allows for autocorrelations and nonstationarity [25]. However, owing to the linear assumption of the ARDL model, it may fail to capture the real relationship between some of the relevant factors in the presence of nonlinear and asymmetric dynamic effects of weather parameters on communicable diseases [26]. Consequently, the NARDL model was used in that it has the advantage of addressing the long- and short-term asymmetric effects over the ARDL model [27]. The NARDL is capable of decomposing the dependent variable into its positive and negative partial sums of increments and decrements in independent variables [27]. When there were nonlinearity and asymmetry in time series, the NARDL model not only deals with the autocorrelations and nonstationarity but also investigates the responses of the dependent variable to positive and negative changes in each of the regressors both in the short and long terms [28]. The NARDL model development includes four procedures [26, 27, 28]: first, test the order of integration. Although the NARDL model can be used irrelevant to the order of integration with the exception that the maximum order is unable to exceed one [26]. Additionally, the nonstationary series can lead to a pseudo regression, and hence the stationarity of the regressors and weather factors and HFRS incidence series was investigated by the augmented Dickey–Fuller test (null hypothesis: There is a unit root present, indicating that the series is nonstationary; alternative hypothesis: There is no unit root present, suggesting that the series is stationary), if the series is showed to be nonstationary (*p* > 0.05), differencing helps obtain a stationary series [26]. Second, investigate the long-term asymmetric cointegration. The bounds test (F statistic) helps judge whether there exists a long-term asymmetric cointegration between weather factors and HFRS incidence series. In the presence of such a relationship, the Wald long-run asymmetry (WLR) and Wald short-run asymmetry (WSR) tests can be applied to explore the long- and short-run asymmetric effects, respectively [27]. Third is estimate effect. Quantify the dynamic multiplier responses of the HFRS incidence series to decreases or increases in weather factors by use of positive and negative partial sum decompositions [27]. Finally, evaluate forecasting ability. To evaluate the predictive performance of the NARDL model by including weather parameters, the dataset from January 2004 to December 2018 served as the training samples, while the rest acted as the testing samples. We then employed several evaluation metrics to compare the predictive capacity between NARDL and ARDL models. These metrics included the mean absolute deviation (MAD), root mean square error (RMSE), mean absolute percentage error (MAPE), mean error rate (MER), and root mean square percentage error (RMSPE) [29]. The comparison was based on the modified Diebold–Mariano (MDM) statistic [30].

The formula of the NARDL is as follows:

(1) $$\begin{array}{c} \begin{array}{c} \text{Log}\left(Y_{t}\right)=a_{0}+\sum_{i=1}^{p_{i}}p_{1i}Y_{t-1}+\sum_{i=0}^{q_{1}}q_{1i}^{+}x_{t-1}^{+}+\sum_{i=0}^{q_{2}}q_{2i}^{-}x_{t-1}^{-}\\ +\sum_{i=1}^{p_{2}}p_{2i}\Delta Y_{t-i}+\sum_{i=0}^{q_{3}}q_{3i}^{+}\Delta x_{t-i}^{+}+\sum_{i=0}^{q_{4}}q_{4i}^{-}\Delta x_{t-i}^{-}+a_{1}\text{month}+a_{2}t+\varepsilon_{t} \end{array}, \end{array}$$

where*Y*_*t*_ is HFRS notification cases, *x* represents the climatic variables, *x*^+^and *x*^−^signify the positive and negative partial sums of increments and decrements in climatic variables, respectively, *p* and *q* stand for the optimal delayed orders of differenced HFRS and weather factors, respectively, month denotes the adjustment for seasonal variable (1, 2,…, 12), and *t* denotes the adjustment for time variable (1, 2,…, 192).

In the analysis, we set the maximum delay orders to 4 months based on the incubation period of 1–6 weeks from hantavirus infection to symptom appearance and the 2-month lag from symptom appearance to clinical diagnosis in China [2]. Subsequently, we determined the optimal delay orders using the Akaike information criterion (AIC). To identify the optimal autocorrelation orders for HFRS, we examined the partial autocorrelogram, which reveals the correlation between present values and past values under specific conditions [31]. Additionally, we included a month parameter in the model to account for seasonal effects. The equation also addressed the long-term trend by adjusting for the time variable. Furthermore, we assessed the stability of the NARDL model by plotting the cumulative sum and cumulative sum of squares [28]. All statistical analyses were conducted using R 4.2.0 (R Development Core Team, Vienna, Austria) and EViews 12 (IHS, Inc., USA). The significance level was set at a two-sided *p* ≤ 0.05.

## 3. Results

### 3.1. Statistical Description

During 2004–2019, 33,565 cases were notified, with an annualized average of 2,098 (5.708 per 100,000 population) and a monthly average of 175 (0.476 per 100,000 population) notification cases. Notably, the maximum number of HFRS cases in 2004 reached 4,171 (10.931 per 100,000 population), which was 3.727 times higher than that in 2017 when it had the lowest level of 1,119 (3.261 per 100,000 population) cases. Collectively, a downward trend in HFRS incidence was seen (average annual percentage change = −6.744%, 95% CI: −13.520%–0.563%), although statistical significance was not strongly supported (*p*=0.07). The decomposition of the season index from January to December was 0.720, 0.551, 0.643, 0.666, 0.994, 1.440, 0.779, 0.449, 0.414, 1.346, 2.629, and 1.370, respectively, revealing a dual seasonal pattern in HFRS incidence. Specifically, there was a strong peak in June and a weaker peak in October–December, while the remaining months had a relative low risk. Additionally, a natural cyclical pattern of approximately 3–5 years was observed in HFRS incidence.

**Figure 1:**
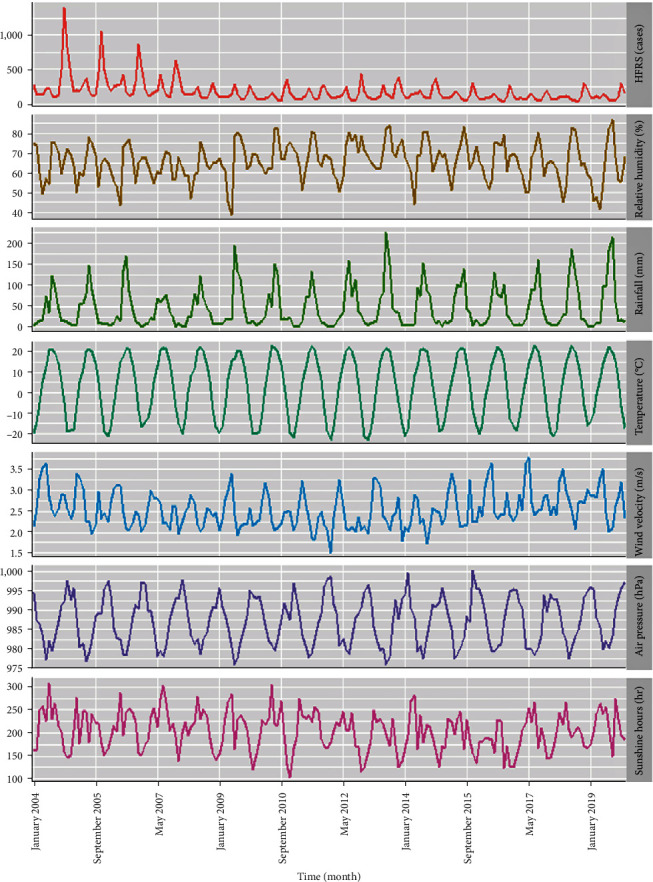
Time series plot indicating the changing patterns of weather parameters and HFRS in Heilongjiang, 2004–2019.

The monthly average relative humidity, aggregate rainfall, average temperature, average wind velocity, average air pressure, and aggregate sunshine hours were 66.07% (59.86%, 71.87%), 65.75 ± 9.37 mm, 5.15°C (−10.94, 15.93°C), 2.47 m/s (2.22, 2.82 m/s), 987.83 hPa (981.71, 992.74 hPa), and 203.88 ± 42.29 hr, respectively (Table 1). It appeared that there was a consistent changing trend between HFRS and wind velocity and air pressure, while a contrasting trend existed between HFRS and relative humidity, aggregate rainfall, temperature, and sunshine hours (Figure 1). Importantly, there was little evidence of strong collinearity among these variables, as indicated by *r*_*s*_ < 0.9 and VIF < 10 (Table 1 and *Supplementary 2*).

### 3.2. Constructing NARDL and ARDL Models

**Figure 2:**
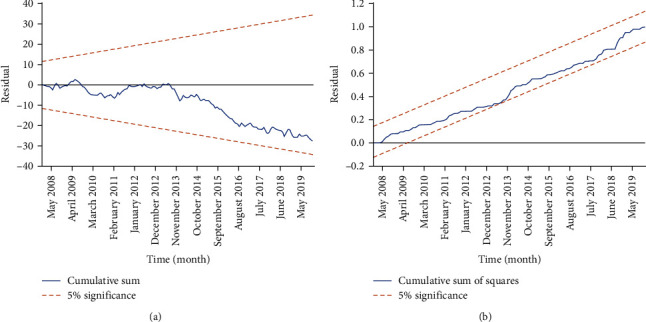
(a, b) Cumulative sum and cumulative sum of squares plot. The residuals at different time points were within the 5% significance level, indicating the stability and adequacy of the best NARDL model.

The augmented Dickey–Fuller test pinpointed that the series for HFRS (*t* = −3.346, *p*=0.014) and air pressure (*t* = −3.328, *p*=0.015) was stationary. However, the series for temperature (*t* = −2.743, *p*=0.069), sunshine hours (*t* = −1.662, *p*=0.449), wind velocity (*t* = −2.030, *p*=0.274), rainfall (*t* = −1.729, *p*=0.415), and relative humidity (*t* = −1.263, *p*=0.646) was nonstationary. After applying first-order differencing, all variables achieved stationarity with *p*-values < 0.001. These results confirmed that the requirement for the model-building process was met. The partial autocorrelogram pinpoints that autocorrelation at a 1-month delay should be entered into the model (*Supplementary 3*). Subsequently, the bounds test revealed that the F-statistic of 6.814 exceeded the critical upper bounds (*I*_0_ = 1.82, *I*_1_ = 2.99), indicating the presence of the long-term cointegration asymmetries between HFRS and climatic parameters. Lastly, a series of NARDL models were built by control for the seasonality, autocorrelation, and secular trend. By comparing all candidates, the NARDL (1, 1, 4, 2, 0, 1, 4, 4, 4, 0, 1, 4, 2) specification emerged as the best-fitting model, yielding the smallest AIC of 0.587 (*Supplementary 4*). The optimal NARDL model included the following parameters: log (HFRS) with a 1-month lag, relative humidity (+) with a 1-month lag and relative humidity (−) with a 4-month lag, rainfall (+) with a 2-month lag and rainfall (−) with a 0-month lag, temperature (+) with a 1-month lag and temperature (−) with a 4-month lag, wind velocity (+) and wind velocity (−) with a 4-month lag, sunshine hours (+) with a 0-month lag and sunshine hours (−) with a 1-month lag, along with air pressure (+) with a 4-month lag and air pressure (−) with a 2-month lag (*Supplementary 5*). Further, the cumulative sum and cumulative sum of squares tests fell within the 5% significance level (Figure 2), validating the stability of the model. Similarly, according to the modeling steps, the ARDL (1, 4, 3, 0, 0, 4, 0) emerged as the optimal model within a wide range of ARDL specifications (the optimal ARDL model included the following parameters: log (HFRS) with a 1-month lag, relative humidity with a 4-month lag, rainfall with a 3-month lag, temperature with a 0-month lag, wind velocity with a 0-month lag, sunshine hours with a 4-month lag, and air pressure with a 0-month lag) (*Supplementary 6* and *Supplementary 7*).

### 3.3. Asymmetric Impacts of Weather Variables on HFRS

**Figure 3:**
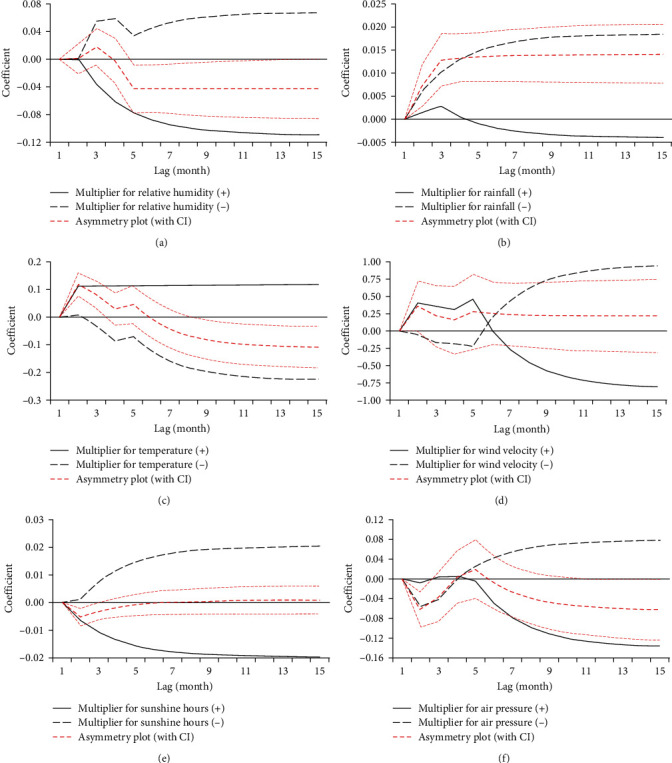
Multiplier asymmetric impact of weather factors on HFRS: (a) multiplier effect for average relative humidity, (b) multiplier effect for aggregate rainfall, (c) multiplier effect for temperature, (d) multiplier effect for average wind velocity, (e) multiplier effect for aggregate sunshine hours, and (f) multiplier effect for average air pressure.

Table 2 presents the results of the WLR and WSR tests and their corresponding effect estimates. Notably, we observed clear evidence of long-term asymmetric impacts of rainfall, wind velocity, and air pressure on HFRS, as well as short-term asymmetric impacts of relative humidity, rainfall, and air pressure on HFRS.  Table 3, on the other hand, reveals that while relative humidity, temperature, and sunshine hours did not exhibit meaningful long-term asymmetry, their long-run coefficients remained significant. Specifically, relative humidity and sunshine hours had negative coefficients, whereas temperature showed the opposite trend. A 1%, 1 hr, and 1 hPa increase in relative humidity, sunshine hours, and air pressure resulted in about 10.9%, 1.9%, and 13.6% decreases in the risk of HFRS transmission, respectively. Conversely, a 1%, 1 mm, and 1 hr decrease in relative humidity, aggregate rainfall, and sunshine hours led to about 6.7%, 1.8%, and 2% decreases in the risk of HFRS transmission, respectively. When temperature increased and decreased by 1°C, the risk of HFRS transmission increased by 11.6% and 22.5%, respectively (the abovementioned increment or decrement in the risk of HFRS transmission represents the cumulative effects of changes in weather factors). Besides, the long-run coefficients of wind velocity, rainfall (+), and air pressure (−) were negative but remarkably insignificant. Although these coefficients may not be practically useful, their directions remain valid. Also, from the data in Table 3, it is apparent that HFRS varied significantly due to positive and negative changes in weather variables at 0−3-month lags over the short term. Specifically, relative humidity (−) and rainfall (−) with a 1-month delay had the strongest negative short-effects, with a 1% and 1 mm decrement leading to 3.2% and 0.3% decrements in the risk of HFRS transmission, respectively. HFRS incidence also varied significantly with positive and negative changes in temperature over the short term. A 1°C increment in temperature at a 0-month delay was correlated with a 11.2% increment in the risk of HFRS transmission, while a 1°C decrement in temperature at a 3-month delay was correlated with 6.4% decrement. An increase of 1 m/s in wind velocity with a 3-month delay, an increase of 1 hPa in air pressure with a 1-month delay, a decrease of 1 m/s in wind velocity with a 1-month delay, and a decrease of 1 hPa in air pressure with a 0-month delay had the strongest short effect, resulting in 53.3%, 5.7%, 45.9%, and 5.6% increases in the risk of HFRS transmission, respectively.  Figure 3 illustrates the asymmetric adjustment patterns of how HFRS accommodates the long-term equilibrium in response to the positive and negative changes in meteorological factors. This corroborated the long- and short-term asymmetric impacts of meteorological factors on HFRS (Figures 3(a), 3(b), 3(c), 3(d), and 3(e)). For example, Figure 3(a) shows that the red dashed line initially rose and then reduced, substantiating a positive short-run asymmetric relationship that eventually turns into a negative long-run asymmetry.

### 3.4. Evaluation of Predictive Accuracy for HFRS Epidemic

**Figure 4:**
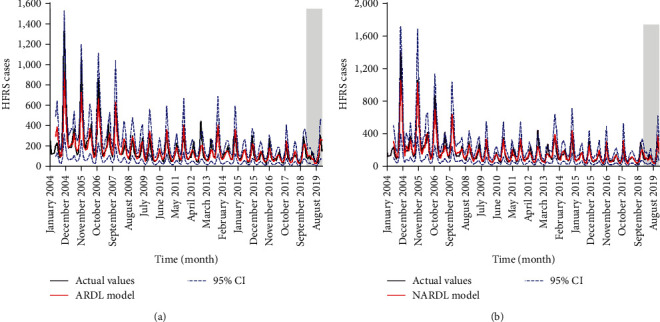
Comparison between the original values and the forecasts generated by (a) the autoregressive distributed lag (ARDL) model, and (b) the nonlinear autoregressive distributed lag (NARDL) model.

This study constructed both ARDL and NARDL models using data spanning from January 2004 to December 2018 and subsequently forecasted the data for January to December 2019. The results of the modeling and forecasting are presented in Figure 4, with the predictive accuracy of both models detailed in Table 4. It was observed that the error metrics from the NARDL model were lower than those from the ARDL model in both mimicking and forecasting parts. The MDM statistics indicated a significant difference between the two models, suggesting that the forecasting performance of the ARDL model was notably inferior to that of the NARDL model. This study also compared the forecasting performance of NARDL and GAM models, finding that the NARDL model had a lower MAPE value (the most commonly used measure of prediction accuracy) than that of the GAM model in both modeling and forecasting phases (*Supplementary 8* and *Supplementary 9*). These results confirmed the suitability and adequacy of NARDL in capturing the dynamic structure of the HFRS epidemic.

## 4. Discussion

This study is the first to use the NARDL model to examine the long- and short-run asymmetric effects of climatic parameters on HFRS by decomposing their changes into positive and negative partial sums and their forecasting performance in Heilongjiang, China. While previous research has examined the relationship between meteorological factors and HFRS [12, 32, 33], few have delved into the effects of sunshine and wind or considered the autocorrelation in the dependent variable. Moreover, the study sheds light on the asymmetric nature of this connection, a dimension that has been largely unexplored. This study highlights the significant long- and short-term asymmetric impacts of meteorological factors on the transmission risk of HFRS at various time lags. The NARDL model, which incorporates these meteorological factors, was found to better capture the dependence structure in HFRS incidence compared to the ARDL and GAM models, as evidenced by its lower MAPE value, which also concurs well with our previous study in Shandong [17]. This underscores the potential of meteorological factors as early indicators for HFRS transmission risk and demonstrates the utility of NARDL model in predicting HFRS epidemics [8]. Similar relationships between weather parameters and other rodent-borne zoonoses (e.g., malaria, dengue fever, cutaneous leishmaniasis, plague, and RossRiver virus infection) have also been observed in previous studies [8, 34, 35]. These findings provide valuable insights for shaping both short- and long-term prevention and control strategies for HFRS.

This study suggested a decreasing trend of HFRS incidence in Heilongjiang from 2004 to 2019, consistent with a previous study in most areas of China [6], but a slight increase in some cities (e.g., Daqing, Songyuan, Tonghua) in recent years [33]. This reduction is attributable to the government's continued endeavor to prevent and control HFRS (e.g., vaccination, rodent control, improved awareness of people, and environmental management) [3, 6]. Additionally, a semiannual seasonal pattern was observed in HFRS morbidity, with a high peak in summer and a low one in winter, similar to the findings in most areas of China [3] and Korea [36]. This pattern may be closely related to weather factors. A preceding study reported a periodic outbreak of 7–12 years in HFRS incidence [6], which we also found in this study, but with a shorter period of 3–5 years. This discrepancy may be associated with the local environment, climate, and socioeconomic factors.

This study indicated a meaningful long-run positive association of temperature with HFRS, meaning that both increases and decreases in temperature led to more HFRS cases. It also found a positive short-run relation when an increment in temperature at a 0-month delay, but a negative short-run relation when a unit decrement in temperature at 0–3 month delays, with the strongest negative effect at a 3-month delay. This short-run association explained the high-risk seasonality of HFRS morbidity, as temperature affects the breeding and survival of rodents, the infectivity of the virus, and the human exposure to rodents in the short and long terms [8]. A previous study showed that Hantaan virus (HTNV) and Seoul virus (SEOV) are the main hantaviruses in Heilongjiang, but SEOV is more prevalent [37]. HTNV-induced HFRS can occur all year round, but mostly in the fall and winter; SEOV-induced HFRS is mainly reported in the spring [38]. This fits well with the high and low peaks of HFRS incidence. The multiplier plot provided evidence that there was a negative short-run impact of temperature on HFRS that turned into a positive long-run effect. However, there is a little evidence of long- and short-run asymmetries between temperature and HFRS. These results match well with several previous studies that found a positive association of temperature with HFRS in Elunchun and Molidawahaner [8]. A prior study indicated the optimal temperature for rodent breeding is 10–25°C [39]. Heilongjiang has a cold climate, so warmer weather may favor the survival of rodents in winter, shorten their maturity period, and increase their contact with humans, thus increasing the risk of HFRS transmission in the long run [7]. On the other hand, for the SEOV dominant areas, a cold climate may affect the food supply and overwintering survival of rodents [12], which may result in a short-run reverse association of temperature with HFRS, but a positive long-term one, as the rodents may depend more on the human living environment and increase the risk of HFRS transmission [12]. However, this finding contradicted with several studies from Guangzhou [9], Shandong [38], Chongqing [40], and the national level of China [41]. This difference may be due to the large variation of environment and climate in the study regions, or the lack of control for the autocorrelation between HFRS series in these studies.

This study found a meaningful long- and short-term asymmetric effect of rainfall on HFRS. From a long-term perspective, aggregate rainfall had a negative effect on HFRS, consistent with previous studies in Shandong [38], Yingshang [42], Jiangsu and Jiaonan [13], Jiamusi and Qiqihar [7]. In China, most HFRS cases occurred in low-lying regions and wetlands [7]. Under such conditions, heavy rainfall can destroy the nests of rodents and reduce the likelihood of rodent–human interaction because of the reduced rodent activity and decreased human exposure in the long term [7]. However, from a short-term view, aggregate rainfall had a positive effect on HFRS, although with a *p*-value of 0.053 in D (rainfall (−)) at a 1-month lag, in agreement with preceding studies [42]. This may be because moist and semimoist soil can enhance vegetation and crops, which provides food for rodents and increases their population density [7]. Short-run heavy rainfall causes flooding, which can force rodents to migrate and spread the disease [43]. However, this finding differed from a study in Heilongjiang that found no relationship between aggregate rainfall and HFRS [10]. This may be because it used a linear ARIMA, which cannot capture the complex relationship.

This study suggested a short-run asymmetric relationship between relative humidity and HFRS but not a long-run one. In the long term, relative humidity had a negative effect on HFRS, consistent with previous studies [12, 13]. This may be because relative humidity is related to rainfall, which can reduce rodent-human contact and the rodent population density, as well as the infectivity and stability of the hantavirus [44]. Interestingly, we found a complex short-run effect of relative humidity on HFRS, with a negative association at a 1-month lag and a positive association at a 3-month lag. A similar reverse effect was reported in a previous study [10], but not in other studies [7, 33, 41]. This difference may be closely related to the specific environment in Heilongjiang. A previous study observed a temporal relationship between the host densities in the third quarter and HFRS in the fourth quarter and a positive association of relative humidity with the host densities in the third quarter [11]. This agreed with the finding of the current study, suggesting that relative humidity can influence the densities of hosts' mites that may play a role in mediating host–host and possibly host–human interaction in the short term.

This work indicated a long-run asymmetry between wind velocity and HFRS instead of a short-run effect. However, the long-run effect was no statistical significance. The short-run wind velocity affected HFRS positively, indicating consistency with other studies [32, 41, 45]. A plausible explanation for this short-run relation is that high wind velocity may favor the dispersal of hantavirus-infected particles in the air, increasing the chance of exposure to them [41]. Besides, wind velocity may also affect the behavior of rodents, which may enhance rodent-human contact. However, the exact mechanism of the positive relation between wind velocity and HFRS is not fully understood and requires further research.

This study showed no long- and short-run asymmetries between sunshine hours and HFRS, but the long-run coefficient was significantly negative, indicating a long-term linear reverse association between them. This finding is not in keeping with previous observational studies, which indicated that sunshine hours affected HFRS positively [32, 41], or that there was no relationship between them [45]. These discrepancies may be associated with different local conditions (e.g., different rodent types, hantavirus serotypes, environments, and climates), or the used models (e.g., ARIMA and GAM), which only focused on the short-run relationship instead of the long-run effect. Sunshine hours may not directly affect HFRS, but it plays an indirect role because the increased exposure to sunlight may result in a reduction in the population of rodents, which can indirectly decrease the risk of HFRS transmission.

This study indicated long- and short-run asymmetries between air pressure and HFRS. In the long term, air pressure had a negative effect on HFRS, which is consistent with other infectious diseases (e.g., pertussis, tuberculosis, and H5N1) [46, 47], although the *p*-value was close to the critical bound. In the short term, air pressure had a positive effect on HFRS, which agrees with several recent studies [32, 45]. The long-term negative relationship between air pressure and HFRS may be explained by the reduced survival and transmission of hantaviruses in an environment with higher air pressure. Hantaviruses are sensitive to temperature and humidity changes, and higher air pressure is often related to lower humidity and higher temperatures, which are detrimental to the survival and transmission of hantaviruses [25]. The short-run positive effect of air pressure on HFRS is unclear and requires further investigation.

There are several limitations that need to be acknowledged in the current study. First, there is a possibility of under-reporting within the passive monitoring system, despite HFRS being a notifiable disease in China. Second, due to the ecological nature of the study, it is not feasible to explore the relationship at an individual level. Third, this study failed to quantify the impact of unmeasured confounding factors. Fourth, this study cannot establish causality between weather variation and HFRS. Lastly, given the existence of spatial heterogeneity and the inconsistency of results across various studies [17, 18], future research should address this heterogeneity. Utilizing the nonlinear panel autoregressive distributed lag (NPARDL) model is recommended, as it has proven to be a valuable tool for analyzing and comprehending the diverse dynamics within heterogeneous panel datasets [48]. By incorporating nonlinear relationships and lag effects, the NPARDL model can effectively capture the complexities of spatial heterogeneity in data analysis [48].

## 5. Conclusions

Investigating both long- and short-term effects can improve the accuracy and reliability of time series analysis and provide researchers with valuable insights for making decisions and forecasts based on a holistic view of the data's temporal dynamics. This study showed that weather parameters had meaningful long- and short-term asymmetric effects on HFRS transmission. Weather factors need to be incorporated into the public health intervention plan for HFRS, especially under the driver of climate change. The NARDL model can simultaneously account for the long- and short-term asymmetries, which makes it more suitable to capture the dynamic dependence of HFRS morbidity than the ARDL model. It can be a promising method to investigate the effects of meteorological parameters on diseases and guide the strategy formulation.

## Figures and Tables

**Table 1:** Summary of monthly HFRS cases and meteorological variables in Heilongjiang, 2004–2019.

Variable	Mean	SD	Min	P_25_	P_50_	P_75_	Max	*p* ^*∗*^	VIF
HFRS cases	174.82	168.98	24.00	83.00	125.50	202.00	1,401.00	<0.001	1.22
Air pressure	65.75	9.37	38.88	59.86	66.07	71.87	86.69	0.211	5.76
Rainfall	46.19	49.70	0.24	9.83	23.73	73.94	225.85	<0.001	6.54
Temperature	2.77	15.01	−23.12	−10.94	5.15	15.93	23.12	<0.001	7.39
Wind velocity	2.54	0.44	1.52	2.22	2.47	2.82	3.77	<0.001	3.38
Air pressure	987.23	6.21	975.92	981.71	987.83	992.74	1,000.30	<0.001	6.16
Sunshine hours	203.88	42.29	103.00	171.56	204.23	231.38	308.32	0.410	2.85

^*∗*^Shapiro–Wilk normality test, *p* < 0.05 indicated the deviation from normality of the data.

**Table 2:** Long- and short-run asymmetric results based on the Wald test.

Variable	Long-run asymmetry		Short-run asymmetry	
	WLR	*p*	WSR	*p*
Relative humidity	1.831	0.069	−1.547	0.001
Rainfall	3.292	0.001	−1.984	0.049
Temperature	−0.285	0.776	0.085	0.932
Wind velocity	−3.271	0.001	−1.414	0.160
Sunshine hours	1.136	0.258	0.934	0.352
Air pressure	−6.453	<0.001	−2.330	0.021

**Table 3:** The resulting results for the long- and short-term coefficients under the optimal NARDL and ARDL models.

ARDL model		
Variable	Coefficient (95% CI)	*p*
Long-run estimate		
Relative humidity	−0.011 (−0.047, 0.025)	0.549
Rainfall	0.005 (−0.003, 0.013)	0.239
Temperature	0.037 (0.013, 0.062)	0.003
Wind velocity	0.106 (−0.258, 0.470)	0.568
Sunshine hours	0.002 (−0.006, 0.010)	0.596
Air pressure	0.005 (0.002, 0.009)	0.006
Short-run estimate		
D (relative humidity)	−0.008 (−0.022, 0.006)	0.287
D (relative humidity), 1-month lag	−0.037 (−0.053, −0.022)	<0.001
D (relative humidity), 2-month lag	−0.032 (−0.046, −0.018)	<0.001
D (relative humidity), 3-month lag	−0.011 (−0.022, −0.0001)	0.049
D (rainfall)	0.001 (−0.002, 0.004)	0.591
D (rainfall), 1-month lag	−0.002 (−0.005, 0.0005)	0.101
D (rainfall), 2-month lag	−0.005 (−0.007, −0.002)	<0.001
D (sunshine hours)	0.0001 (−0.002, 0.002)	0.947
D (sunshine hours), 1-month lag	−0.005 (−0.008, −0.002)	0.001
D (sunshine hours), 2-month lag	−0.007 (−0.010, −0.005)	<0.001
D (sunshine hours), 3-month lag	−0.002 (−0.004, 0.0003)	0.088
Time	−0.004 (−0.005, −0.002)	<0.001
Seasonality	−0.005 (−0.036, 0.025)	0.732

NARDL model		
Variable	Coefficient (95% CI)	*p*
Long-run estimate		
Relative humidity (+)	−0.109 (−0.178, −0.040)	0.003
Relative humidity (−)	−0.067 (−0.135, 0.001)	0.057
Rainfall (+)	−0.004 (−0.019, 0.012)	0.618
Rainfall (−)	−0.018 (−0.033, −0.002)	0.028
Temperature (+)	0.116 (0.041, 0.191)	0.003
Temperature (−)	0.225 (0.141, 0.308)	<0.001
Wind velocity (+)	−0.777 (−1.929, 0.375)	0.188
Wind velocity (−)	−0.973 (−2.390, 0.445)	0.181
Sunshine hours (+)	−0.019 (−0.030, −0.008)	0.001
Sunshine hours (−)	−0.020 (−0.032, −0.008)	0.001
Air pressure (+)	−0.136 (−0.280, 0.008)	0.067
Air pressure (−)	−0.073 (−0.202, 0.056)	0.267
Short-run estimate		
D (relative humidity (+))	0.001 (−0.023, 0.026)	0.921
D (relative humidity (+))	0.001 (−0.023, 0.026)	0.921
D (relative humidity (−))	0.001 (−0.018, 0.019)	0.952
D (relative humidity (−)), 1-month lag	−0.032 (−0.054, −0.010)	0.004
D (relative humidity(−)), 2-month lag	0.000 (−0.020, 0.020)	1.000
D (relative humidity (−)), 3-month lag	0.027 (0.011, 0.044)	0.002
D (rainfall (+))	0.001 (−0.002, 0.005)	0.457
D (rainfall(−)), 1-month lag	0.003 (0.000, 0.006)	0.053
D (temperature (+))	0.112 (0.077, 0.147)	<0.001
D (temperature (−))	−0.007 (−0.043, 0.029)	0.700
D (temperature (−)), 1-month lag	−0.042 (−0.072, −0.011)	0.010
D (temperature (−)), 2-month lag	−0.014 (−0.047, 0.018)	0.398
D (temperature (−)), 3-month lag	−0.064 (−0.094, −0.034)	<0.001
D (wind velocity (+))	0.397 (0.074, 0.720)	0.017
D (wind velocity (+)), 1-month lag	0.366 (−0.032, 0.763)	0.073
D (wind velocity (+)), 2-month lag	0.342 (0.000, 0.685)	0.052
D (wind velocity (+)), 3-month lag	0.533 (0.215, 0.851)	0.001
D (wind velocity (−))	0.033 (−0.319, 0.386)	0.853
D (wind velocity (−)), 1-month lag	0.459 (0.055, 0.862)	0.028
D (wind velocity (−)), 2-month lag	0.393 (0.051, 0.736)	0.026
D (wind velocity (−)), 3-month lag	0.424 (0.146, 0.702)	0.003
D (sunshine hours (+))	−0.001 (−0.005, 0.002)	0.506
D (air pressure (+))	−0.008 (−0.041, 0.026)	0.660
D (air pressure (+)), 1-month lag	0.057 (0.017, 0.096)	0.006
D (air pressure (+)), 2-month lag	0.050 (0.013, 0.086)	0.008
D (air pressure (+)), 3-month lag	0.039 (0.009, 0.070)	0.013
D (air pressure (−))	0.056 (0.020, 0.092)	0.003
D (air pressure (−)), 1-month lag	0.030 (−0.004, 0.065)	0.087
Time	0.135 (0.013, 0.257)	0.032
Seasonality	−0.064 (−0.105, −0.023)	0.003

**Table 4:** Comparison of the mimicking and forecasted abilities between ARDL and NARDL models.

Models	Mimicking part					Forecasting part				
	MAD	MAPE	RMSE	MER	RMSPE	MAD	MAPE	RMSE	MER	RMSPE
ARDL	43.141	0.241	73.394	0.241	0.316	39.6411	0.406	48.622	0.360	0.467
NARDL	35.232	0.207	55.600	0.197	0.263	25.598	0.213	35.288	0.233	0.283
MDM	2.411	2.658	1.482	—	2.269	4.629	10.370	4.120	—	9.197
*P*	0.017	0.009	0.140	—	0.025	<0.001	<0.001	<0.001	—	<0.001

## Data Availability

All data used in this study were publicly available.

## Supplementary Materials

Supplementary 1 Geographical distribution of Heilongjiang (Created by ArcGIS 10.8). *Note*. The basic geographic information data of China were downloaded from the National Geomatics Center of China (Available at: https://www.ngcc.cn/. Accessed on 12 March, 2024).

Supplementary 2 Spearman's rank correlation among meteorological factors.

Supplementary 3 Partial autocorrelogram for the differenced HFRS series. It was shown that the partial autocorrelation at a l-month lag is just touching the significance bounds, meaning that there existed the first-order autocorrelation between HFRS series.

Supplementary 4 The top 20 NARDL models with a lower Akaike information criterion (AIC) among candidates. It was found that the NARDL (1, 1, 4, 2, 0, 1, 4, 4, 4, 0, 1, 4, 2) specification has the lowest AIC, and thus it should be identified as the optimal model.

Supplementary 5 Estimated parameters for the selected best possible NARDL (1, 1, 4, 2, 0, 1, 4, 4, 4, 0, 1, 4, 2) model.

Supplementary 6 The top 20 ARDL models with a lower Akaike information criterion (AIC) among candidates. It was found that the ARDL (1, 4, 3, 0, 0, 4, 0) specification has the lowest AIC, and thus it should be identified as the optimal model.

Supplementary 7 Estimated parameters for the selected best possible ARDL (1, 4, 3, 0, 0, 4, 0) model.

Supplementary 8 The resulting effective degrees of freedom and the statistical test results of the GAM model.

Supplementary 9 Comparison of the mimicking and forecasted abilities between ARDL, NARDL, and GAM models.
