# Supplementary material for: Long- and Short-Run Asymmetric Effects of Meteorological Parameters on Hemorrhagic Fever with Renal Syndrome in Heilongjiang: A Population-Based Retrospective Study
Source: Transbound Emerg Dis. 2024 Jul 30;2024:6080321. doi: 10.1155/2024/6080321 (PMC12016769; doi:10.1155/2024/6080321)
Supplement: Supplementary 1 — Geographical distribution of Heilongjiang (Created by ArcGIS 10.8). Note. The basic geographic information data of China were downloaded from the National Geomatics Center of China (Available at: https://www.ngcc.cn/. Accessed on 12 March, 2024). [file 6080321.f1.docx]

**Asymmetric effects in the context of disease epidemics[**[**1-5**](#_ENREF_1)**]**

Asymmetric effects play a crucial role in understanding the dynamics of diseases, especially when analyzing them using the NARDL model. This model allows researchers to capture non-linear relationships and asymmetries that may exist in the data, providing a more comprehensive understanding of the impact of diseases on populations.

First, diseases often exhibit non-linear patterns in their spread and impact on individuals. Traditional linear models may fail to capture the complex interactions between different factors that influence the progression of a disease. By using the NARDL model, researchers can account for non-linear relationships and asymmetries in the data, allowing for a more accurate representation of the dynamics of diseases. This is particularly important when studying diseases that have varying effects on different population groups or in different stages of the disease.

Second, asymmetries in the effects of diseases can have significant implications for public health interventions and policies. For example, certain diseases may have a more severe impact on vulnerable populations, such as the elderly or individuals with pre-existing health conditions. By using the NARDL model to analyze the data, researchers can identify these asymmetries and tailor interventions to target the most affected groups. This can help to improve the effectiveness of public health strategies and ensure that resources are allocated efficiently to those who need them the most.

Finally, understanding asymmetric effects can also provide valuable insights into the underlying mechanisms of diseases. By identifying the factors that contribute to asymmetries in the data, researchers can gain a deeper understanding of the biological, social, and environmental factors that influence the spread and severity of diseases. This knowledge can inform the development of new treatments and prevention strategies, ultimately leading to better outcomes for patients and communities.


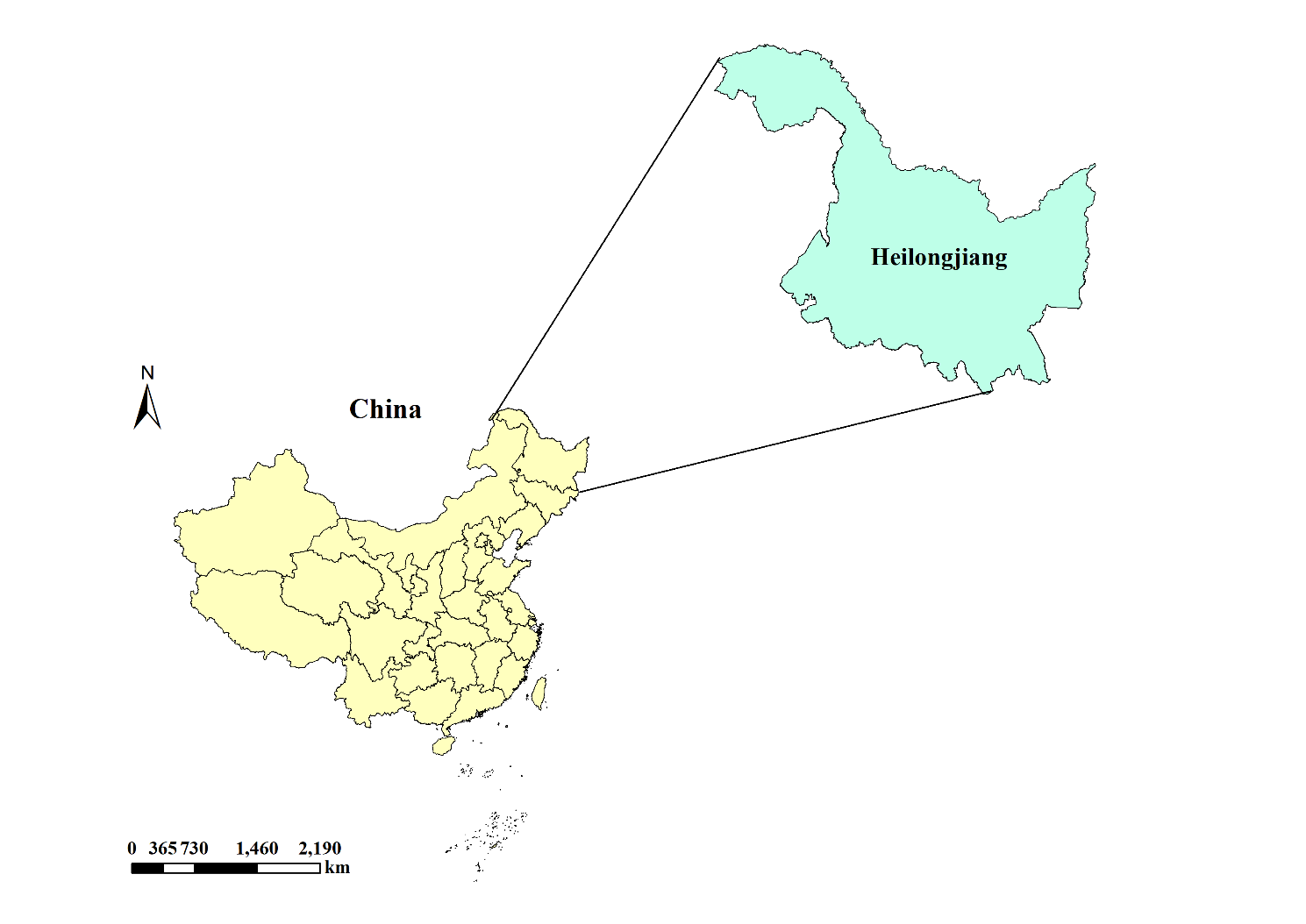


Figure S1. Geographical distribution of Heilongjiang (Created by ArcGIS 10.8). Note: The basic geographic information data of China were downloaded from the National Geomatics Center of China (Available at: http://www.ngcc.cn/ngcc/. Accessed on 12 March, 2024).

**References**

1. Das P. Cointegration, Error Correction and Vector Autoregression. In: Das P, editor. Econometrics in Theory and Practice: Analysis of Cross Section, Time Series and Panel Data with Stata 151. Singapore: Springer Singapore; 2019. p. 367-416.

2. Diaye MA, Ho SH, Oueghlissi R. ESG performance and economic growth: a panel co-integration analysis. Empirica. 2021:1-24.

3. Allen DE, Mcaleer M. A Nonlinear Autoregressive Distributed Lag (NARDL) Analysis of the FTSE and S&P500 Indexes. Risks. 2021;9:1-20.

4. Sharaf M, Shahen A. Asymmetric impact of real effective exchange rate changes on domestic output revisited: evidence from Egypt. EconStor Open Access Articles and Book Chapters. 2023:2-15.

5. Shin Y, Yu B, Greenwoodnimmo M. Modelling Asymmetric Cointegration and Dynamic Multipliers in a Nonlinear ARDL Framework. Social Science Electronic Publishing. 2014:281-314.
