# Supplementary figures and images for: Long- and Short-Run Asymmetric Effects of Meteorological Parameters on Hemorrhagic Fever with Renal Syndrome in Heilongjiang: A Population-Based Retrospective Study
Source: Transbound Emerg Dis. 2024 Jul 30;2024:6080321. doi: 10.1155/2024/6080321 (PMC12016769; doi:10.1155/2024/6080321)

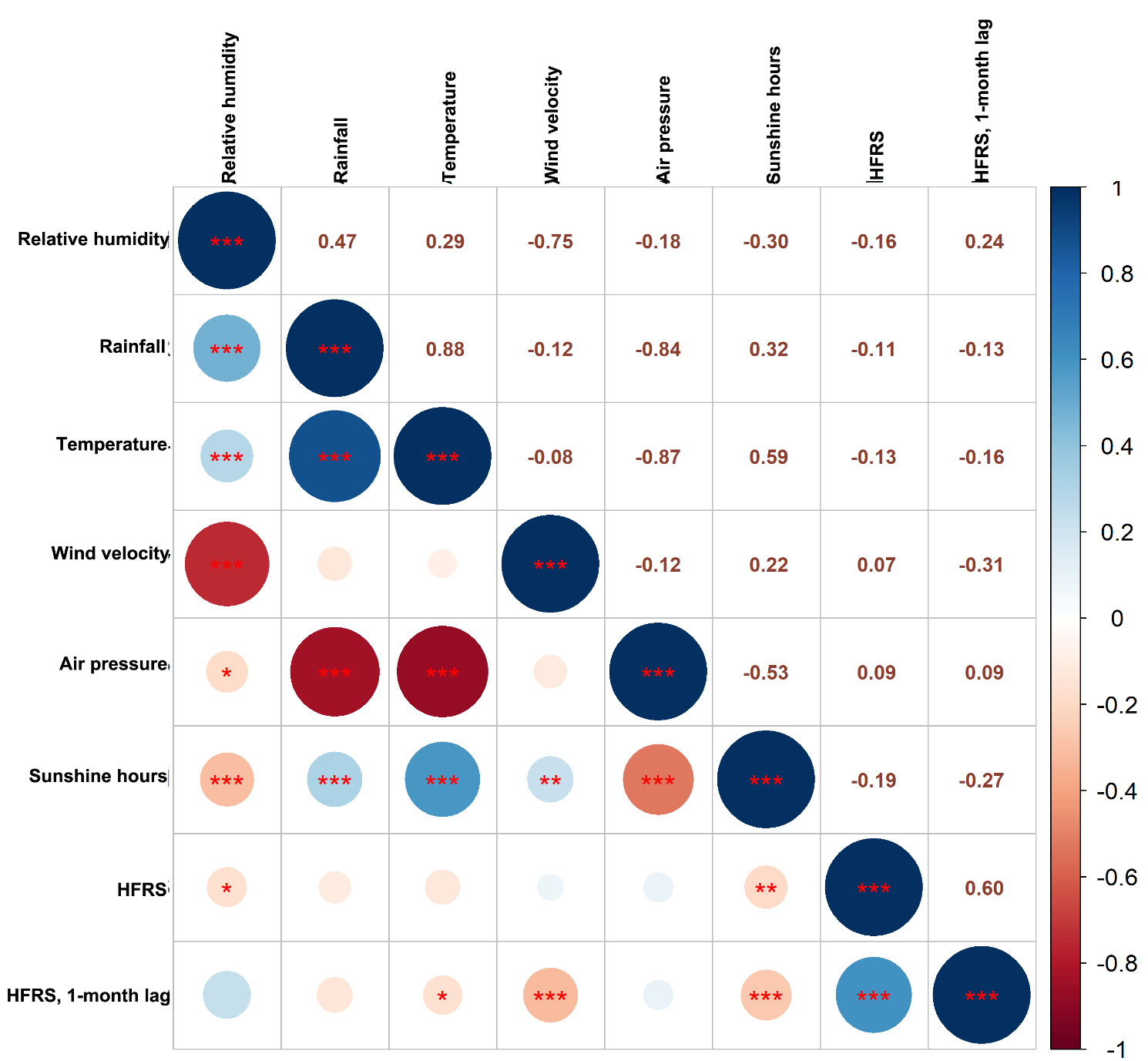


Figure S2. Spearman’s rank correlation among meteorological factors.

Supplement: Supplementary 2 — Spearman's rank correlation among meteorological factors. [file 6080321.f2.docx]
