# Supplementary material for: Long- and Short-Run Asymmetric Effects of Meteorological Parameters on Hemorrhagic Fever with Renal Syndrome in Heilongjiang: A Population-Based Retrospective Study
Source: Transbound Emerg Dis. 2024 Jul 30;2024:6080321. doi: 10.1155/2024/6080321 (PMC12016769; doi:10.1155/2024/6080321)
Supplement: Supplementary 3 — Partial autocorrelogram for the differenced HFRS series. It was shown that the partial autocorrelation at a l-month lag is just touching the significance bounds, meaning that there existed the first-order autocorrelation between HFRS series. [file 6080321.f3.docx]

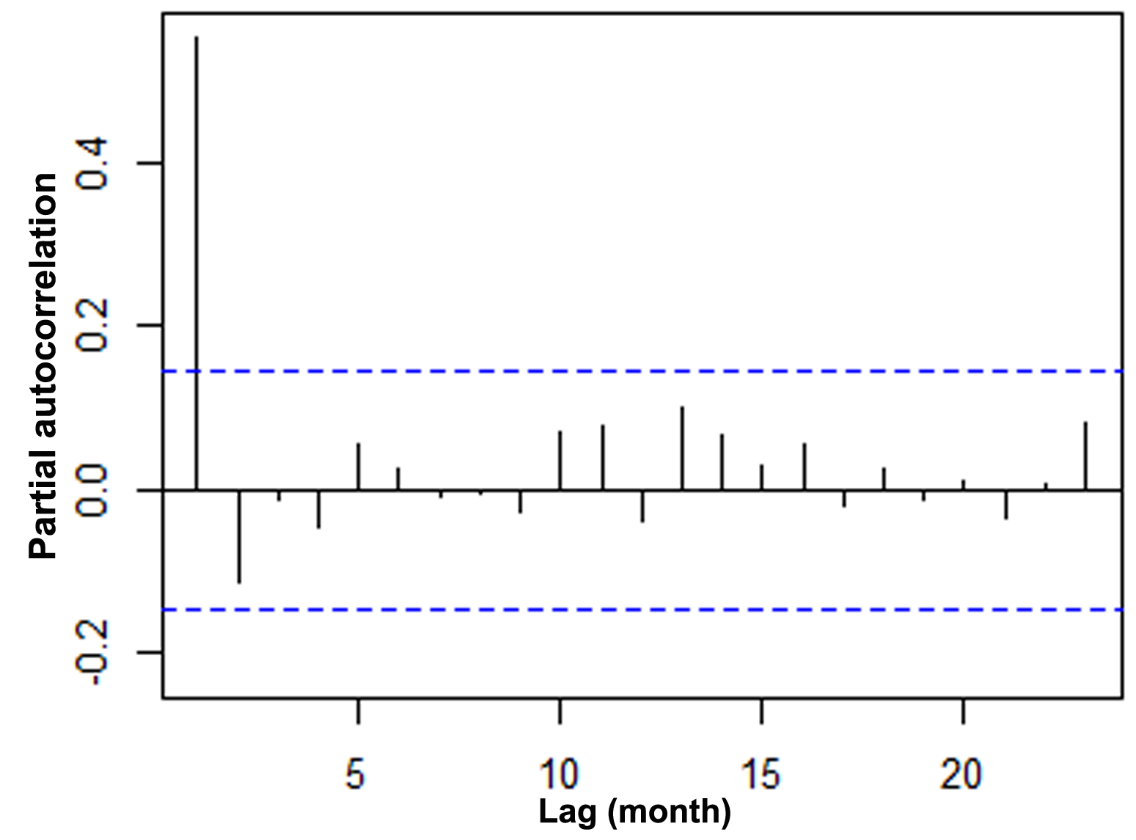


Figure S3. Partial autocorrelogram for the differenced HFRS series. It was shown that the partial autocorrelation at a l-month lag is just touching the significance bounds, meaning that there existed the first-order autocorrelation between HFRS series.
