# Supplementary material for: Long- and Short-Run Asymmetric Effects of Meteorological Parameters on Hemorrhagic Fever with Renal Syndrome in Heilongjiang: A Population-Based Retrospective Study
Source: Transbound Emerg Dis. 2024 Jul 30;2024:6080321. doi: 10.1155/2024/6080321 (PMC12016769; doi:10.1155/2024/6080321)
Supplement: Supplementary 5 — Estimated parameters for the selected best possible NARDL (1, 1, 4, 2, 0, 1, 4, 4, 4, 0, 1, 4, 2) model. [file 6080321.f5.docx]

**Table S1.** Estimated parameters for the selected best possible NARDL(1, 1, 4, 2, 0, 1, 4, 4, 4, 0, 1, 4, 2) model

| Variable | Coefficient | Std. Error | t-Statistic | *p* |
| --- | --- | --- | --- | --- |
| HFRS, 1-month lag | 0.653 | 0.053 | 12.381 | <0.001 |
| Relative humidity(+) | 0.001 | 0.013 | 0.099 | 0.921 |
| Relative humidity(+), 1-month lag | -0.039 | 0.013 | -3.100 | 0.002 |
| Relative humidity(-) | 0.001 | 0.010 | 0.061 | 0.952 |
| Relative humidity(-), 1-month lag | -0.056 | 0.011 | -5.324 | <0.001 |
| Relative humidity(-), 2-month lag | 0.032 | 0.010 | 3.074 | 0.003 |
| Relative humidity(-), 3-month lag | 0.027 | 0.010 | 2.662 | 0.009 |
| Relative humidity(-), 4-month lag | -0.027 | 0.008 | -3.231 | 0.002 |
| Rainfall(+) | 0.001 | 0.002 | 0.745 | 0.457 |
| Rainfall(+), 1-month lag | 0.000 | 0.002 | 0.088 | 0.930 |
| Rainfall(+), 2-month lag | -0.003 | 0.002 | -1.947 | 0.053 |
| Rainfall(-) | -0.006 | 0.002 | -2.549 | 0.012 |
| Temperature(+) | 0.112 | 0.018 | 6.320 | <0.001 |
| Temperature(+),1-month lag | -0.072 | 0.016 | -4.587 | <0.001 |
| Temperature(-) | -0.007 | 0.018 | -0.386 | 0.700 |
| Temperature(-), 1-month lag | 0.044 | 0.021 | 2.063 | 0.041 |
| Temperature(-), 2-month lag | 0.027 | 0.020 | 1.356 | 0.177 |
| Temperature(-), 3-month lag | -0.050 | 0.021 | -2.356 | 0.020 |
| Temperature(-), 4-month lag | 0.064 | 0.015 | 4.195 | <0.001 |
| Wind velocity(+) | 0.397 | 0.165 | 2.407 | 0.017 |
| Wind velocity(+), 1-month lag | -0.301 | 0.212 | -1.423 | 0.157 |
| Wind velocity(+), 2-month lag | -0.023 | 0.195 | -0.120 | 0.905 |
| Wind velocity(+), 3-month lag | 0.191 | 0.191 | 0.999 | 0.319 |
| Wind velocity(+), 4-month lag | -0.533 | 0.162 | -3.282 | 0.001 |
| Wind velocity(-) | 0.033 | 0.180 | 0.185 | 0.853 |
| Wind velocity(-), 1-month lag | 0.087 | 0.192 | 0.454 | 0.650 |
| Wind velocity(-), 2-month lag | -0.065 | 0.156 | -0.420 | 0.675 |
| Wind velocity(-), 3-month lag | 0.031 | 0.160 | 0.194 | 0.846 |
| Wind velocity(-), 4-month lag | -0.424 | 0.142 | -2.994 | 0.003 |
| Sunshine hours(+) | -0.007 | 0.002 | -4.046 | <0.001 |
| Sunshine hours(-) | -0.001 | 0.002 | -0.667 | 0.506 |
| Sunshine hours(-), 1-month lag | -0.006 | 0.002 | -3.033 | 0.003 |
| Air pressure(+) | -0.008 | 0.017 | -0.441 | 0.660 |
| Air pressure(+), 1-month lag | 0.017 | 0.018 | 0.934 | 0.352 |
| Air pressure(+), 2-month lag | -0.007 | 0.017 | -0.408 | 0.684 |
| Air pressure(+), 3-month lag | -0.010 | 0.017 | -0.600 | 0.550 |
| Air pressure(+), 4-month lag | -0.039 | 0.016 | -2.509 | 0.013 |
| Air pressure(-) | 0.056 | 0.018 | 3.032 | 0.003 |
| Air pressure(-), 1-month lag | -0.051 | 0.020 | -2.552 | 0.012 |
| Air pressure(-), 2-month lag | -0.030 | 0.018 | -1.722 | 0.087 |
| t | 0.135 | 0.062 | 2.162 | 0.032 |
| Seasonality | -0.064 | 0.021 | -3.077 | 0.003 |

NARDL, nonlinear autoregressive distributed lag model; HFRS, Hemorrhagic fever with renal syndrome.
