# Supplementary material for: Long- and Short-Run Asymmetric Effects of Meteorological Parameters on Hemorrhagic Fever with Renal Syndrome in Heilongjiang: A Population-Based Retrospective Study
Source: Transbound Emerg Dis. 2024 Jul 30;2024:6080321. doi: 10.1155/2024/6080321 (PMC12016769; doi:10.1155/2024/6080321)
Supplement: Supplementary 6 — The top 20 ARDL models with a lower Akaike information criterion (AIC) among candidates. It was found that the ARDL (1, 4, 3, 0, 0, 4, 0) specification has the lowest AIC, and thus it should be identified as the optimal model. [file 6080321.f6.docx]

**Figure S5.** The top 20 ARDL models with a lower Akaike information criterion (AIC) among candidates. It was found that the ARDL(1, 4, 3, 0, 0, 4, 0) specification has the lowest AIC, and thus it should be identified as the optimal model.
