# Supplementary material for: Long- and Short-Run Asymmetric Effects of Meteorological Parameters on Hemorrhagic Fever with Renal Syndrome in Heilongjiang: A Population-Based Retrospective Study
Source: Transbound Emerg Dis. 2024 Jul 30;2024:6080321. doi: 10.1155/2024/6080321 (PMC12016769; doi:10.1155/2024/6080321)
Supplement: Supplementary 7 — Estimated parameters for the selected best possible ARDL (1, 4, 3, 0, 0, 4, 0) model. [file 6080321.f7.docx]

**Table S2.** Estimated parameters for the selected best possible ARDL(1, 4, 3, 0, 0, 4, 0) model

| Variable | Coefficient | Std. Error | t-Statistic | *p* |
| --- | --- | --- | --- | --- |
| HFRS, 1-month lag | 0.462 | 0.067 | 6.913 | <0.001 |
| Relative humidity | -0.008 | 0.007 | -1.068 | 0.287 |
| Relative humidity, 1-month lag | -0.036 | 0.006 | -5.738 | <0.001 |
| Relative humidity, 2-month lag | 0.005 | 0.007 | 0.713 | 0.477 |
| Relative humidity, 3-month lag | 0.021 | 0.007 | 2.992 | 0.003 |
| Relative humidity, 4-month lag | 0.011 | 0.006 | 1.986 | 0.049 |
| Rainfall | 0.001 | 0.001 | 0.538 | 0.591 |
| Rainfall, 1-month lag | -0.001 | 0.002 | -0.393 | 0.695 |
| Rainfall, 2-month lag | -0.002 | 0.001 | -1.568 | 0.119 |
| Rainfall, 3-month lag | 0.005 | 0.001 | 3.912 | <0.001 |
| Temperature | 0.020 | 0.006 | 3.435 | <0.001 |
| Wind velocity | 0.057 | 0.099 | 0.577 | 0.565 |
| Sunshine hours | 0.000 | 0.001 | 0.066 | 0.947 |
| Sunshine hours, 1-month lag | -0.004 | 0.001 | -2.952 | 0.004 |
| Sunshine hours, 2-month lag | -0.002 | 0.001 | -1.794 | 0.075 |
| Sunshine hours, 3-month lag | 0.005 | 0.001 | 4.374 | <0.001 |
| Sunshine hours, 4-month lag | 0.002 | 0.001 | 1.717 | 0.088 |
| Air pressure | 0.003 | 0.001 | 2.521 | 0.013 |
| t | -0.004 | 0.001 | -5.399 | <0.001 |
| Seasonality | -0.005 | 0.016 | -0.343 | 0.732 |

ARDL, autoregressive distributed lag model; HFRS, Hemorrhagic fever with renal syndrome.
