# Supplementary material for: Long- and Short-Run Asymmetric Effects of Meteorological Parameters on Hemorrhagic Fever with Renal Syndrome in Heilongjiang: A Population-Based Retrospective Study
Source: Transbound Emerg Dis. 2024 Jul 30;2024:6080321. doi: 10.1155/2024/6080321 (PMC12016769; doi:10.1155/2024/6080321)
Supplement: Supplementary 8 — The resulting effective degrees of freedom and the statistical test results of the GAM model. [file 6080321.f8.docx]

**Table S3.** The resulting effective degrees of freedom and the statistical test results of the GAM model

| Variables | EDF | Ref.df | F | *p* |
| --- | --- | --- | --- | --- |
| s(relative humidity) | 1.000 | 1.000 | 3.717 | 0.056 |
| s(rainfall) | 1.000 | 1.000 | 3.382 | 0.068 |
| s(temperature) | 6.465 | 7.571 | 4.827 | <0.001 |
| s(wind velocity) | 1.463 | 1.797 | 0.663 | 0.596 |
| s(air pressure) | 4.163 | 5.214 | 3.374 | 0.001 |
| s(sunshine hours) | 1.000 | 1.000 | 14.044 | <0.001 |
| s(t) | 5.485 | 6.640 | 15.379 | <0.001 |

EDF, effective degrees of freedom; seasonality was also adjusted in the equation. In this study, to further highlight the advantages of the NARDL model, we additionally constructed the commonly used GAM model and compared its predictive performance with the NARDL model. The specific details of the GAM model are as follows. In our study, it was assumed that the monthly HFRS cases followed a quasi-Poisson distribution, and a model linking the logarithm of expected HFRS cases with meteorological variables was constructed using a GAM model with a Gaussian distribution family. The optimal degrees of freedom (d_f_) for the spline function were estimated using Generalized Cross-Validation (GCV) criteria. A previous study has underscored the importance of incorporating factors such as changes in population immunity, autocorrelations, various lag structures and relationships, seasonality, and long-term trends when conducting time series analysis[[1](#_ENREF_1)]. Therefore, in the process of building the GAM model, the model was further calibrated by accounting for temporal trends and seasonality as confounding factors.

**References**

1. Imai C, Armstrong B, Chalabi Z, Mangtani P, Hashizume M. Time series regression model for infectious disease and weather. Environ Res. 2015;142:319-27.
