# Supplementary material for: Long- and Short-Run Asymmetric Effects of Meteorological Parameters on Hemorrhagic Fever with Renal Syndrome in Heilongjiang: A Population-Based Retrospective Study
Source: Transbound Emerg Dis. 2024 Jul 30;2024:6080321. doi: 10.1155/2024/6080321 (PMC12016769; doi:10.1155/2024/6080321)
Supplement: Supplementary 9 — Comparison of the mimicking and forecasted abilities between ARDL, NARDL, and GAM models. [file 6080321.f9.docx]

**Table S4.** Comparison of the mimicking and forecasted abilities between ARDL, NARDL, and GAM models

| Models | Mimicking part | | | | |  | Forecasting part | | | | |
| --- | --- | --- | --- | --- | --- | --- | --- | --- | --- | --- | --- |
|  | MAD | MAPE | RMSE | MER | RMSPE |  | MAD | MAPE | RMSE | MER | RMSPE |
| ARDL | 43.141 | 0.241 | 73.394 | 0.241 | 0.316 |  | 39.6411 | 0.406 | 48.622 | 0.360 | 0.467 |
| NARDL | 35.232 | 0.207 | 55.600 | 0.197 | 0.263 |  | 25.598 | 0.213 | 35.288 | 0.233 | 0.283 |
| GAM | 43.598 | 0.277 | 65.376 | 0.243 | 0.368 |  | 24.184 | 0.261 | 34.020 | 0.220 | 0.363 |

ARDL, autoregressive distributed lag model; NARDL, nonlinear autoregressive distributed lag model; GAM, generalized additive model; MAD, mean absolute deviation; MAPE, mean absolute percentage error; RMSE, root mean square error; MER, mean error rate; RMSPE, root mean square percentage error.
